# The relationship between male sexual signals, cognitive performance, and mating success in stickleback fish

**DOI:** 10.1002/ece3.3091

**Published:** 2017-06-15

**Authors:** Ross Minter, Jason Keagy, Robin M. Tinghitella

**Affiliations:** ^1^ Department of Biological Sciences University of Denver Denver CO USA; ^2^ Department of Animal Biology University of Illinois Urbana‐Champaign IL USA

**Keywords:** cognition, detour‐reaching task, female choice, inhibitory control, learning, sexual selection, sexual signal, threespine stickleback

## Abstract

Cognitive ability varies dramatically among individuals, yet the manner in which this variation correlates with reproduction has rarely been investigated. Here, we ask (1) do male sexual signals reflect their cognitive ability, and (2) is cognitive ability associated with male mating success? Specifically, we presented threespine sticklebacks (*Gasterosteus aculeatus*) with a detour‐reaching task to assess initial inhibitory control. Fish that performed better were those who solved the detour‐reaching task, solved it faster, and required fewer attempts to solve. We then reexamined males’ performance on this task over several days to assess learning ability in this context. We next measured sexual signals (coloration, nest area, and courtship vigor) and asked whether they reveal information about these male cognitive abilities. Finally, we examined whether success at attracting a female is associated with male cognition. After controlling for the strong effect of neophobia, we found that no measured sexual signals were associated with initial inhibitory control. Sexual signals were also not associated with change in performance on the detour‐reaching task over time (learning). However, females preferred mating with males who had better initial inhibitory control. We speculate that inhibitory control is a critical trait for male sticklebacks. In this system, males perform all parental care, but must avoid eating their own fry which closely resemble their prey items. Therefore, males with better inhibitory control may be more likely to successfully raise their offspring to independence. Our research adds to a growing list of mating systems and taxa in which cognition is important for measures related to fitness.

## 1 INTRODUCTION

Animal courtship displays can be strikingly elaborate. They may contain several sequential steps, occur in multiple modalities (e.g., visual, acoustic, and tactile signals), involve the integration of morphological and behavioral signals, and be context dependent. For example, peacock spiders synchronously use motion displays, body ornamentation, and vibrations in their courtship display (Girard, Kasumovic, & Elias, 2011). Proper coordination of these courtship signals is essential for them to operate as an effective display. Recent findings suggest that cognitive ability may underlie both the production and assessment of elaborate displays (Boogert, Fawcett, & Lefebvre, 2011; Boogert, Giraldeau, & Lefebvre, 2008; Keagy, Savard, & Borgia, 2012; Ryan, Akre, & Kirkpatrick, 2009).

Cognition is broadly defined as the neurological manner in which animals acquire, process, retain, and use information (Dukas, 2004; Shettleworth, 2001). Cognition aids in vital elements of survival such as foraging and predator avoidance (Dukas, 1998) and can buffer animals from environmental stressors, lowering mortality (Sol, Székely, Liker, & Lefebvre, 2007). Cognition also has important effects on the process of sexual selection (Boogert, Fawcett, et al., 2011; Ryan et al., 2009). Previous research on the role of cognition in mate choice has primarily focused on learning in the contexts of courtship behavior (Beecher & Brenowitz, 2005; Ejima, Smith, Lucas, Levine, & Griffith, 2005; Hollis, Pharr, Dumas, Britton, & Field, 1997; King & West, 1983; Maggio, Maggio, & Whitney, 1983), and sexual trait preferences (Dukas, 2008; Galef & White, 1998; Hebets, 2003; reviewed in Ryan et al., 2009). More recently, evidence is growing that cognition may assist males in obtaining mates. For instance, male bowerbirds and sage grouse that more strategically adapt their courtship behavior in response to female signaling have higher mating success (Patricelli, Coleman, & Borgia, 2006; Patricelli & Krakauer, 2010; Patricelli, Krakauer, & Mcelreath, 2011; Patricelli, Uy, Walsh, & Borgia, 2002). Furthermore, superior foragers may signal their foraging ability to females via exaggerated sexual signals in carotenoid‐dependent signaling systems (Endler, 1980; Karino, Shinjo, & Sato, 2007; Mateos‐Gonzalez, Quesada, & Senar, 2011). In general, we expect female preferences for mates with particular enhanced cognitive abilities to evolve if females gain either direct and/or indirect benefits when mating with them (Boogert, Fawcett, et al., 2011; Keagy, Savard, & Borgia, 2009).

Although in its infancy, the study of the role of cognition in sexual selection has begun to develop as a field (see review in Boogert, Fawcett, et al., 2011). Song complexity, a sexual signal, has been linked to performance on a novel foraging task in zebra finches (Boogert et al., 2008), suggesting that performance on a foraging task may be communicated through sexual signals. In wild‐caught song sparrows, song repertoire size correlates with detour reaching, which is related to inhibitory control (Boogert, Anderson, Peters, Searcy, & Nowicki, 2011). Male guppies that learned a maze more quickly (potentially indicating foraging abilities) also produced higher quality carotenoid signals (Karino et al., 2007), which are often under sexual selection (Endler, 1980). In a separate study, female guppies preferred males who learned mazes quickly, although in this study, learning speed was not associated with carotenoid signals (Shohet & Watt, 2009). Finally, male satin bowerbird mating success is positively associated with their problem‐solving performance and aggregate measures of their cognitive ability (Keagy, Savard, & Borgia, 2011; Keagy et al., 2009). Females appear to select these high performing mates by integrating information about several behavioral display traits (Keagy et al., 2012).

Despite growing evidence that females choose mates on the basis of cognitive traits, this is certainly not always the case. For instance, song complexity and repertoire size do not always positively correlate with cognitive performance on different cognitive tasks (Anderson et al., 2016; Boogert, Anderson, et al., 2011; Sewall et al., 2013; Templeton, Laland, & Boogert, 2014; perhaps because of developmental trade‐offs (Sewall, Soha, Peters, & Nowicki, 2013). Similarly, female spotted bowerbirds do not appear to select males with better general cognitive abilities or performance on single tasks like barrier removal or shape discrimination (Isden, Panayi, Dingle, & Madden, 2013). Finally, starlings raised with developmental stress had a reduced sexual signal (song bouts), but performance on a foraging task did not differ (Farrell, Weaver, An, & MacDougall‐Shackleton, 2012).

**Figure 1:**
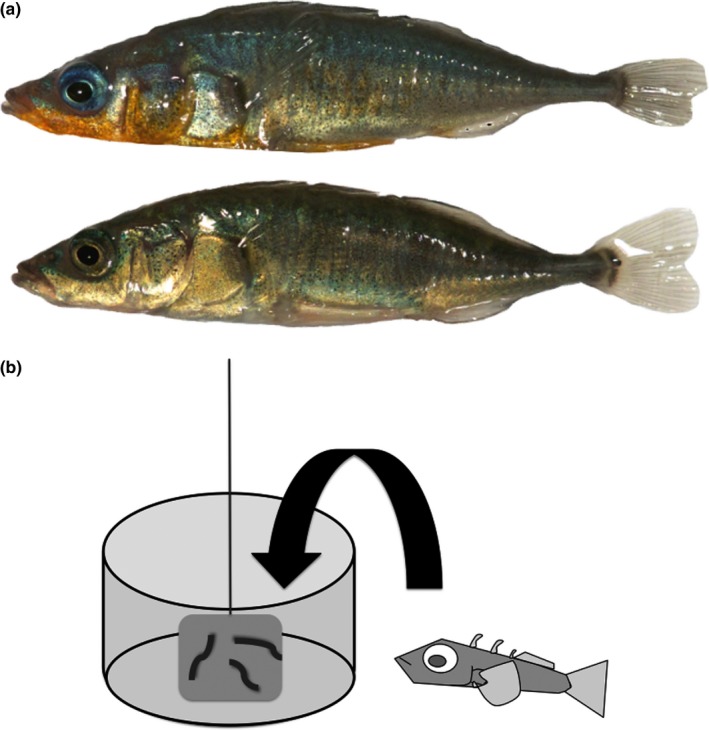
Male threespine sticklebacks and the detour‐reaching task apparatus. (a) Representative males with extensive red coloration (top) and reduced red coloration (bottom). (b) Sticklebacks accessed the food reward (bloodworms on the outside of a clear bag, represented by dark grey lines) by swimming above and into the cut‐out circle on the top of the clear, cylindrical barrier

Here, we use threespine stickleback (*Gasterosteus aculeatus*; Figure 1), to investigate whether male sexual signals reflect their cognitive ability, and whether male cognitive ability is associated with male ability to attract females. Threespine sticklebacks are small fish with obligate male paternal care in most populations (including the one we studied). Stickleback courtship is sequential and complex (Bell & Foster, 1994; Nagel & Schluter, 1998) and females assess multiple sexual signals (courtship vigor: Vamosi & Schluter, 1999; red throat color: Milinski & Bakker, 1990; Scott, 2004; Tinghitella, Lehto, & Minter, 2015; blue eye color: Rowland, 1994; the interaction of throat and eye color: Flamarique, Bergstrom, Cheng, & Reimchen, 2013; Rowe, Baube, Loew, & Phillips, 2004; features of nests: Candolin & Voigt, 1998; Sargent, 1982; Östlund‐Nilsson & Holmlund, 2003). Stickleback males must appropriately respond to female signals to progress through the courtship sequence. In addition, adult sticklebacks are predators on stickleback eggs and fry (Foster, Garcia, & Town, 1988; Hynes, 1950; Whoriskey & FitzGerald, 1985) and parental males must resist eating their offspring to ensure fitness. These features led us to study inhibitory control, the ability to inhibit an ineffective prepotent behavior or ignore irrelevant stimuli when attempting to achieve a goal (Boogert, Anderson, et al., 2011; Hauser, 1999; MacLean et al., 2014). Inhibitory control is a crucial and well‐studied component of executive function and is often critical for decision‐making and problem‐solving (Amici, Aureli, & Call, 2008; Chow, Leaver, Wang, & Lea, 2017; Hopewell & Leaver, 2008; Kralik, Hauser, & Zimlicki, 2002; MacLean et al., 2014; Meulman, Seed, & Mann, 2013).

We consider two questions: (1) Do male threespine stickleback sexual signals indicate male cognitive ability? And, (2) Does male cognitive ability predict acceptance by females as mates (i.e., male mating success, a component of male fitness)? We presented males a detour‐reaching task to assess inhibitory control four times over a period of seven days. We assessed males’ initial ability to maneuver around a clear cylinder to reach a food reward rather than attempting to swim through the cylinder (initial inhibitory control) and their improvement over time (learning via operant conditioning, Staddon & Cerutti, 2003). We then measured male sexual signals and their acceptance by females as mates. We made the following predictions. First, we anticipated that males with higher quality sexual signals, such as redder throats and more intense blue eyes, would have better measures of inhibitory control and learning. Second, we expected females to choose males who had better inhibitory control and/or were better learners as mates, perhaps because these males are more likely to successfully raise offspring to independence.

## 2 METHODS

All research was conducted with approval from The University of Denver's IACUC (2013‐0004). Collection and transport permits were obtained from the Washington Department of Fish and Wildlife (14‐078). We collected reproductive threespine sticklebacks from the Chehalis River (46° 58′ 42″N, 123° 28′ 46″W) in SW Washington, USA, in April 2014. We transported fish to the University of Denver and housed them in single sex groups in 110‐L (77 × 32 × 48 cm) or 284‐L (123 × 47 × 54 cm) home tanks at a density of approximately one fish per 5‐L. We fed all individuals in home tanks a mixture of defrosted brine shrimp (*Artemia* sp) and defrosted bloodworms (chironomid larvae) and just brine shrimp on alternating days. We kept fish in a temperature and photoperiod controlled room set to 17°C and 15:9‐hr light:dark cycle in the beginning of the experiment. Broad‐spectrum (400–900 nm) Sylvania Octron Eco 5000K fluorescent lights illuminated the room. We adjusted the light:dark cycle throughout the breeding season to track conditions in SW Washington. Before trials began, we relocated individuals from their home tanks to randomly assigned visually isolated 110‐L (77 × 32 × 48 cm) experimental tanks. Each tank contained an artificial plant, a gravel pack (crushed coral in a nylon casing, used to maintain water quality), and a nesting tray (17 × 11 × 3 cm) filled with sand and covered by half of a flower pot (15 × 16 × 7 cm). We provided 5 g of nesting material (*Ceratophyllum demursum*) to each male. These items mimicked their natural environment in a way that encouraged males to build nests. We conducted cognition testing and mate choice trials in the experimental tanks. Detour‐reaching and mate choice trials were conducted from June to August of 2014. Conducting both detour‐reaching and mate choice trials during the reproductive season allowed us to capture the sexual signals on which females base their mating decisions. Sticklebacks do not express sexual signals outside of the breeding season. In addition, a male's inhibitory control during the mating season (as opposed to the non‐breeding season) is likely more relevant to female fitness as males with good inhibitory control may be more likely to successfully raise offspring, giving females both indirect and direct benefits.

### 2.1 Detour‐reaching task

Before presenting males with the detour‐reaching task, we allowed fish to acclimate to the experimental tanks for 24 hr (day zero). We did not feed fish during this 24‐hr period to increase motivation to reach the food reward. The detour‐reaching task was presented on days one, two, four, and seven, and always followed a 24‐hr break from food. This sequence of four trials allowed us to assess whether learning occurred. Before each trial began, we lowered an opaque divider into the tank, blocking the fish's view of the barrier and food reward as they were placed into the tank. We used a transparent plastic container (11.5 cm diameter base, 7 cm tall) with a 9.5 cm diameter opening on the top as our barrier (Figure 1). In the center of the clear plastic container, we suspended a small clear plastic bag (3 × 2.5 cm) containing bloodworms and water. This bag increased the saliency of the food reward, which consisted of three bloodworms attached to the outside of the bag using petroleum jelly.

We began observations when the opaque divider was removed. To retrieve the food reward, the fish needed to swim above and into the cylinder through the opening, whereas a fish's initial response tended to be to swim directly into the transparent wall of the cylinder (55 of 58 males tested, 94.8%). The trial concluded when the fish entered the cylinder or after 10 min, whichever came first. Once the trial ended, we removed the cylinder and food reward bag. We fed fish brine shrimp (ad libitum) as a daily source of food when they were in experimental tanks, except on days preceding a trial. Additionally, fish that did not enter the cylinder and receive the food reward were given three bloodworms. In this way, all fish were fed equal numbers of bloodworms. Feeding not associated with the detour‐reaching task always occurred at least one hour after the trial.

During each detour‐reaching trial, we recorded whether the fish entered the cylinder, the number of attempts each fish made to access the food, as well as the time to enter the cylinder, using the event recorder JWatcher (Blumstein, Evans, & Daniel, 2006). We coded an attempt as any occurrence of a fish physically contacting the barrier. In our analyses, we used the inverse of attempts (hereafter “entries per attempts”), which resulted in a variable ranging from (nearly) 0 to 1; individuals that entered the cylinder without first physically contacting the barrier (1 entry/0 attempts) were given a score of 1. We assigned the maximum possible trial time (10 min = 600 s) to all fish that did not enter the cylinder. We also quantified the number of detour‐reaching trials until first cylinder entry; if a fish never successfully entered the cylinder after the four trials, it was assigned a score of a 5. Detour‐reaching tasks were presented to 58 males in total.

### 2.2 Mate choice

Males could begin nest construction immediately upon placement into their experimental tanks. To prompt males to construct and maintain nests (which are necessary to assess female mate choice in sticklebacks), we introduced randomly chosen gravid females into experimental tanks daily for 10 min (hereafter referred to as “enticement”). Males did not see the same gravid female on each daily enticement, so any effect of the quality of the females used in enticement was spread randomly across males and repeated presentations. On days when a detour‐reaching task was presented (days one, two, four, and seven after introduction), enticement always occurred after the presentation. If a male had completed nest building, enticement took place with a female enclosed in a jar to prevent spawning. We considered a nest to be “under construction” if the male had begun to fasten down sand or plants with spiggin (a glue‐like protein males produce for nest building). We considered nests complete when an opening and exit were clearly visible (Wootton, 1976). There is a good deal of natural variation in the time it takes males to build a nest; males that built nests took an average of 7 days (mean ± *SE*; 7.44 ± 0.98) to do so. To maximize the number of males who could be used in mate choice trials, if a male had begun but not completed building a nest by day 7, he was given up to an additional 7 days to complete nest‐building. Additionally, males that did not begin to nest during the 7 days over which detour‐reaching tasks were presented were removed from experimental tanks, but then given a second opportunity to nest after all other males had completed detour‐reaching trials. One male was inadvertently offered a third opportunity to nest. Twenty‐seven of 58 males nested in this experiment.

To assess female choice, we conducted no‐choice mating trials with methods commonly used by multiple stickleback laboratories (Head, Price, & Boughman, 2009; Nagel & Schluter, 1998; Tinghitella, Weigel, Head, & Boughman, 2013). Courtship trials were conducted as soon as males had completed nest building. For each trial, a gravid female was placed into an opaque cylinder with a manually operated exit within his tank. Following a 2‐min acclimation period, the female was released into the tank and we recorded behaviors related to mate choice using JWatcher. Trials lasted 20 min or until the female entered the nest, whichever came first. Entering the males’ nest is the final stage of courtship and occurs immediately before egg deposition. If females entered nests, we carefully removed them before they could deposit their eggs. We used each male in mate choice trials two times; there were two exceptions because two males did not maintain their nests long enough to be paired with a second gravid female. Males underwent their second mate choice trial soon after the first (mean ± *SE*: 1.42 ± 0.22 days) and were enticed on the days on which mate choice trials were not conducted. We used females up to two times except for two females (one who was used three times, the other four). We never paired a male with the same female twice. Similar to previous work, we allowed males and females to rest for at least 2 hr between mating trials (Kozak, Head, Lackey, & Boughman, 2013; Tinghitella et al., 2013). Following each mating trial, we photographed males and females with a Canon Powershot G15 under standardized conditions. We weighed males and females to the nearest hundredth of a gram (Scout Pro SP202). We completed 52 courtship trials with 27 males and 38 females.

### 2.3 Sexual signals

Color scores were assessed for all nesting males immediately before and after each mate choice trial. We assessed male throat color area and intensity, and eye color intensity using a scale of 0–5 with half‐point increments (zero indicating no color and 5 indicating maximum color area or intensity) using standardized methods in which the male is briefly held in hand and compared to a set of photograph standards for each component of color (Boughman, 2001, 2007; Lackey & Boughman, 2013; Lewandowski & Boughman, 2008; Tinghitella et al., 2013, 2015). Color scores reliably match reflectance data (Albert, Millar, & Schluter, 2007; Boughman, 2007; Wedekind, Meyer, Frischknecht, Niggli, & Pfander, 1998). We additively combined (equally weighted) throat area and intensity into one measure that we call “throat score” (after Lackey & Boughman, 2013). Because males were used in two mating trials each, we took the grand mean of the four color measurements (before and after scores for each of two mate choice trials) for use in models assessing the relationship between color sexual signals and detour‐reaching performance. We used the average of the before and after color scores for individual trials in models assessing relationships between detour‐reaching performance and female choice.

In addition to examining male coloration, we measured nest area and courtship vigor. Larger nests may indicate readiness to invest energy toward reproduction (McKaye, Louda, & Stauffer, 1990; Östlund‐Nilsson & Holmlund, 2003; Soler, Møller, & Soler, 1998). To measure nest area, we photographed nests using a Canon Powershot G15 equipped with a Canon WP‐D48 waterproof case. We photographed the nests immediately following the males’ last mate choice trials. We measured area in nest photographs using ImageJ version 1.47 (http://rsb.info.nih.gov/ij/), outlining the perimeter of the nest and establishing scale using a ruler visible in each photograph. To quantify male courtship vigor, we summed all male courtship behaviors directed toward the female and divided by trial duration (in seconds); the mean of courtship vigor from both mate choice trials was used in models assessing the relationship between courtship vigor and detour‐reaching performance.

### 2.4 Statistical analysis

All statistical analyses were conducted in R (R Core Team, 2016, version v3.3.2). For all analyses, we arcsine square root transformed entries per attempts, and log transformed time to solve to improve normality. In tests where multiple response variables were tested against the same variable set, we adjusted significance values for these multiple comparisons with Bonferonni's corrections.

#### 2.4.1 Test for learning

To assess whether learning occurred across repeated detour‐reaching trials, we measured how male performance [entering the cylinder, (arcsine square root transformed) entries per attempts, and (log transformed) time to enter] changed over the four trials using a mixed models approach. We used three separate models (one with each cognitive performance measure as a response). Each model included trial number (1, 2, 3, and 4) as a fixed effect and estimated different intercepts and slopes for the relationship between time and performance for each male (i.e., random intercepts and slopes for male identity). We used a binomial generalized linear mixed model for entries [using “glmer” function in the “lme4” library (Bates, Maechler, Bolker, & Walker, 2013)] and a linear mixed model for the other two response variables (using the “lmer” function in the “lme4” library). We compared each model to a reduced null model that had no fixed effect and included only male identity as a random effect and determined statistical significance using chi‐squared tests.

#### 2.4.2 Cognition measures

We had three measures of detour‐reaching task performance for each trial: entering the cylinder (yes/no), (arcsine square root transformed) entries per attempts, and (log transformed) time to enter. In our analyses of the relationships between cognitive performance, sexual signals, and fitness components, we were interested both in initial performance and learning (improvement over time).

As a variable reduction technique, we first performed principal components analysis (PCA) on the three standardized performance measures (*z*‐scores) from the first trial (Lande & Arnold, 1983) in R using the “prcomp” function in the “stats” library. Mixing binary and continuous variables in a PCA is acceptable when used to summarize variation in a set of variables, as we do here (Everitt & Hothorn, 2011). The first principal component (PC1_detour‐reaching_) explained much of the variance (72%) of all three performance measures, which loaded very evenly (Table 1a). Thus, higher values of PC1_detour‐reaching_ describe fish who were better at the detour‐reaching task according to all three performance measures.

**Table 1:** Principal Components Analysis for variable reduction of initial detour‐reaching performance variables (a) and learning slopes measures (b)

(a) Trial 1 Variable (*N* = 58)	PC1 Eigenvector	PC2 Eigenvector	PC3 Eigenvector
Enter (yes/no)	0.58	−0.45	−0.67
Entries/Attempts	0.59	−0.34	0.74
Time to Enter	−0.56	−0.83	0.07
Eigenvalue	1.47	0.69	0.61
% Variance	71.9	15.7	12.4

(b) Learning Slopes Variable (*N* = 58)	PC1 Eigenvector	PC2 Eigenvector	
Change in Entries/Attempts	0.71	0.71	
Change in Time to Enter	−0.71	0.71	
Eigenvalue	1.21	0.74	
% Variance	72.7	27.3	

In each case, we used the first principal component (PC1) in further analyses that assessed relationships between cognition, sexual signals, and female mate choice.

**Figure 2:**
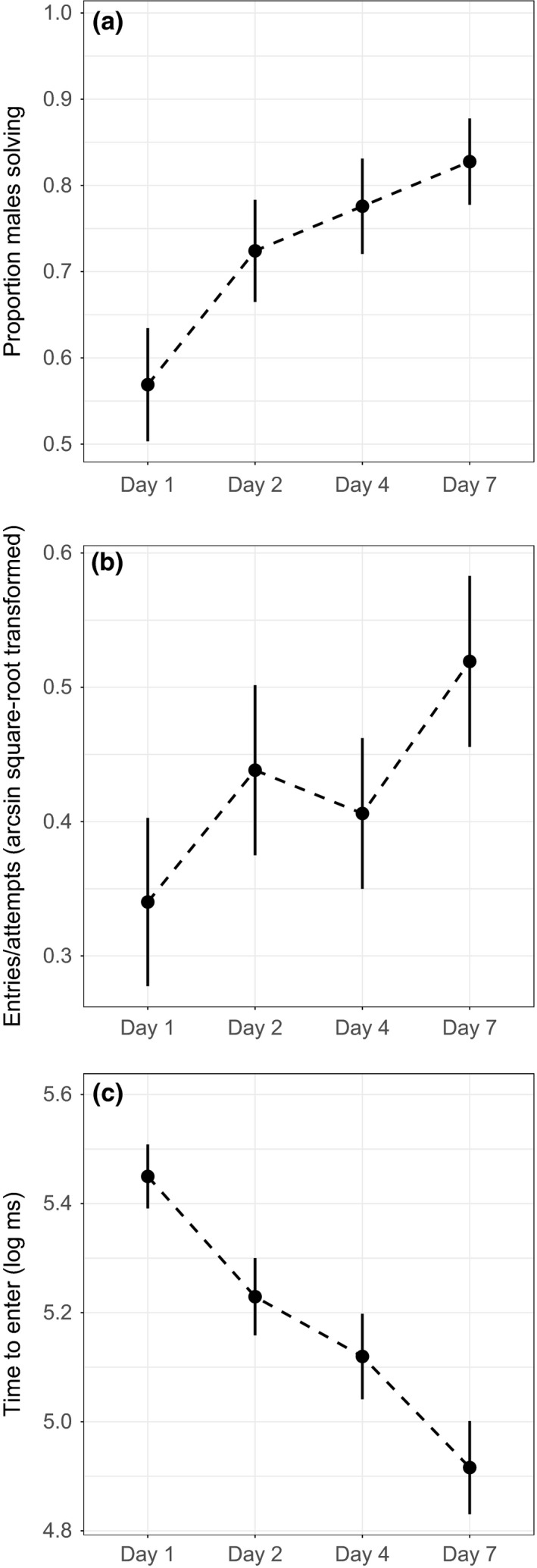
Male performance on detour‐reaching task over time. Change over time for (a) the proportion of males entering the cylinder, (b) (arcsine square root transformed) entries per attempts, and (c) (log transformed) time to enter the cylinder. Plotted are the mean ± *SE*

On average, males improved in our three measures of detour‐reaching task performance over time, especially if only the first and last trials were considered (see 3, Figure 2). Therefore, we initially quantified learning with three different measures: the number of presentations until first entry of the cylinder, the change in entries per attempts, and the change in time to enter the cylinder. To obtain the latter two measures of learning, we performed linear regressions of (arcsine square root transformed) entries per attempts and (log transformed) time to enter on trial number (1, 2, 3, and 4) and used the slopes from these regressions. Better learners would have positive slopes in the models with entries per attempts and negative slopes in the models with time to enter. Next, we again performed PCA as a variable reduction technique on the two slope variables (change in entries per attempts and change in time to enter). PC1_learning_ explained 73% of the variance of the two slope variables, which loaded evenly (Table 1b). Fish with higher PC1_learning_ scores are better learners (i.e., with each successive trial, they have more entries per attempts and take less time to enter the cylinder). We retained number of trials to enter the first time as a separate variable because while the two slope variables are fairly highly correlated with each other, neither is correlated with number of trials to enter and so this variable appears to be independent (see Table S1).

#### 2.4.3 Sexual signals and cognition

We assessed the relationship between PC1_detour‐reaching_ and male color sexual signals in a multiple regression. The model initially included throat color score, eye intensity, and their interaction as main effects. We then removed the nonsignificant interaction from the model. We also included as covariates three potentially confounding variables that could affect performance: male mass, neophobia (time to first contact the barrier on the first presentation), and the number of days it took males to nest. Male mass may influence energetic needs and hence motivation, neophobia may affect how males interacted with the detour‐reaching task, and when males built nests could be related to reproductive state during the detour‐reaching task trials. We then used two multiple regressions to assess relationships between PC1_learning_ and color sexual signals, and number of trials to enter and color sexual signals. The main effects and covariates were the same as above, and, again, we removed nonsignificant interactions from the models. Only nesting males (*N* = 27) were included in models describing the relationship between color sexual signals and PC1_detour‐reaching_, PC1_learning_, and number of trials to solve.

To assess whether the noncolor sexual signals of courtship vigor and nest area were related to cognitive performance, we performed multiple regression models with the males that built nests and underwent mate choice trials (*N* = 27). The main effects and covariates were the same as above, and, again, we removed nonsignificant interactions from the models.

Our analyses revealed that neophobia was strongly correlated with PC1_detour‐reaching_ but neither of the learning variables (see 3). This could result in multicollinearity in our models relating PC1_detour‐reaching_ to variables related to fitness (described below). Therefore, we calculated the residuals of a regression of PC1_detour‐reaching_ on neophobia. By removing the effect of neophobia on PC1_detour‐reaching_, these residuals should better reflect the cognitive component of detour‐reaching task performance, which has been interpreted as inhibitory control (for simplicity, we will refer to these residuals as “inhibitory control”). Positive residuals represent individuals who were better at the detour‐reaching task than predicted by their neophobia. We verified our results from the models described above with multiple regressions substituting inhibitory control for PC1_detour‐reaching_.

#### 2.4.4 Mate choice and cognition

Female acceptance of a male during mate choice trials did not depend on trial order (χ^*2*^ = 0.09, *df* = 1, *p* = .76). We used generalized linear mixed models to address whether females preferred males with better cognitive performance. We performed these analyses in R using the “glmer” function in the “lme4” library. The binomial response variable was whether or not the female entered the nest. In some stickleback populations, acceptance of a male (entering a nest) is rare, and thus, female choice is assessed with other measures (Head et al., 2009; Kozak, Reisland, & Boughman, 2009). In our study, females entered nests in 54% of trials, making entering the nest a useful metric of mate acceptance. In separate models, we included inhibitory control, PC1_learning_, and number of trials to enter as our fixed effects. Male and female IDs were included as random effects because males and females were often used more than once in different mate choice trials. We included male sexual signals (throat color score, eye intensity, nest area, and courtship vigor), mass, neophobia, and time to build a nest as covariates in these models. We included the sexual signals because of a lack of relationship between the sexual signals and cognition (see 3). Therefore, with this analysis, we are asking whether cognition predicts residual variance in female preference not explained by sexual signals that do not appear to be related to cognition. If cognition does predict residual variance, this would suggest that there may be unmeasured traits females could be using to assess male cognitive ability. We included time to build a nest as a covariate because males that built a nest quickly may be more motivated to breed and had fewer opportunities for physical interaction with females during daily enticement prior to mate choice tests (see description of enticement above). We included neophobia due to the possibility that males who were neophobic may have been less likely to approach the female initially. Finally, we compared each model (with one fixed effect) to a reduced null model that included all covariates and random effects (no fixed effects) and determined statistical significance using chi‐squared tests.

## 3 RESULTS

### 3.1 Change in barrier test performance over time

Frequency of entering the cylinder increased (generalized linear mixed model: χ^*2*^ = 23.05*, df = *3, *p* = 3.94 × 10^−5^; Figure 2a) and time to enter decreased over the four trials (linear mixed model: χ^*2*^ = 51.41*, df = *3, *p* = 4.01 × 10^−11^; Figure 2c). There was not a significant change across all four trials for entries per attempts (linear mixed model: χ^*2*^ = 6.43*, df = *3, *p* = .09; Figure 2b), although the first and last trials did differ (paired *t*‐test: *t*_*57*_ = −2.08, *p* = .042).

### 3.2 Sexual signals and cognition

No color sexual signals were associated with PC1_detour‐reaching_ scores, PC1_learning_ scores or the number of trials required to successfully enter the cylinder (Table 2). Neither nest size nor courtship vigor predicted any cognition measures (Tables 3 and 4). However, males who were less neophobic (made contact with the cylinder more quickly upon first presentation) were better at the detour‐reaching task the first time they encountered it (Table 2, 3, 4).

**Table 2:** Relationship between male color signals and cognitive performance

Cognition Measure	Fixed Effect/Covariate	*t*	*df*	*p*	Adjusted *p*
PC1_detour‐reaching_	Body Mass	0.21	21	.838	1.000
	**Neophobia**	−**2.80**	**21**	**.011**	**.032**
	Nesting Time	−0.05	21	.957	1.000
	Throat Color Score	−0.59	21	.560	1.000
	Eye Intensity	1.29	21	.212	.637
PC1_learning_	Body Mass	−0.75	21	.459	1.000
	Neophobia	1.64	21	.116	.348
	Nesting Time	−0.80	21	.435	1.000
	Throat Color Score	−0.28	21	.783	1.000
	Eye Intensity	1.46	21	.160	.480
Number of Presentations to Enter	Body Mass	0.36	21	.723	1.000
	Neophobia	0.94	21	.358	1.000
	Nesting Time	1.30	21	.206	.619
	Throat Color Score	0.42	21	.680	1.000
	Eye Intensity	−2.20	21	.039	.118

We considered three measures of cognition: initial detour‐reaching performance, learning, and number of presentations to enter the cylinder. Male body mass, neophobia, and how many days it took males to nest were included as covariates. Significant effects after Bonferroni's correction are highlighted in bold.

**Table 3:** Relationships between nest area and cognitive performance

Cognition measure	Fixed effect/Covariate	*t*	*df*	*p*	Adjusted *P*
PC1_detour‐reaching_	Body mass	0.59	22	.559	1.000
	**Neophobia**	−**2.68**	**22**	**.014**	**.041**
	Nesting time	0.17	22	.865	1.000
	Nest area	−0.48	22	.634	1.000
PC1_learning_	Body mass	−0.44	22	.667	1.000
	Neophobia	1.35	22	.191	.573
	Nesting time	−0.55	22	.591	1.000
	Nest area	0.07	22	.945	1.000
Number of presentations to enter	Body mass	−0.29	22	.775	1.000
	Neophobia	0.93	22	.362	1.000
	Nesting time	0.61	22	.550	1.000
	Nest area	0.65	22	.524	1.000

Cognition measures are as in Table 2. Male body mass, neophobia, and nesting time were included as covariates. Significant effects after Bonferroni's correction are highlighted in bold.

**Table 4:** Relationships between courtship vigor and cognitive performance

Cognition measure	Fixed effect/Covariate	*t*	*df*	*p*	Adjusted *p*
PC1_detour‐reaching_	Body mass	0.33	22	.747	1.000
	**Neophobia**	−**2.87**	**22**	**.009**	**.027**
	Nest time	−0.03	22	.974	1.000
	Courtship vigor	−0.32	22	.755	1.000
PC1_learning_	Body mass	−0.04	22	.970	1.000
	Neophobia	1.35	22	.190	.570
	Nest time	−0.50	22	.625	1.000
	Courtship vigor	1.04	22	.312	.624
Number of presentations to enter	Body mass	−0.22	22	.828	1.000
	Neophobia	1.17	22	.257	.771
	Nest time	0.88	22	.386	1.000
	Courtship vigor	−0.33	22	.744	1.000

Cognition measures are as in Table 2. Male body mass, neophobia, and nesting time were included as covariates. Significant effects after Bonferroni's correction are highlighted in bold.

### 3.3 Female preference and cognition

**Figure 3:**
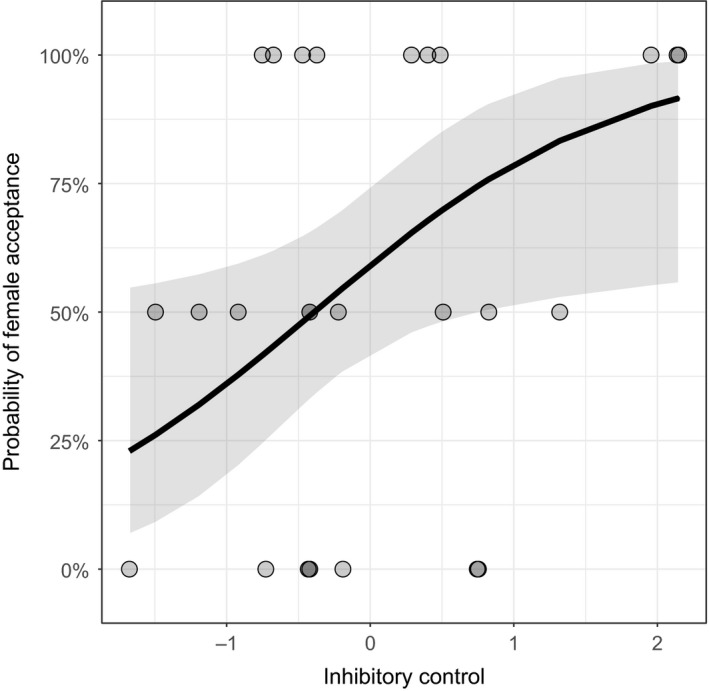
Female choice and cognition. Males who were accepted by females as mates had better inhibitory control. Shown here is the line indicating the marginal effect of inhibitory control, with remaining covariates (throat and eye color, nest area, courtship vigor, body mass, neophobia, and days to build a nest) set to their means. The 95% confidence interval is indicated by the gray shading on either side of the line. This model also included female ID and male ID as random factors. Data points, however, are the means for each male across his trials (usually 2, see 2). The data points are partially transparent; darker regions indicate more overlap between individual points

We found that one measure of male cognition, inhibitory control, predicted female acceptance of males in a model that also contained sexual signals, neophobia, mass, and time to build a nest as covariates. Males who were accepted by females as mates had better inhibitory control (Table 5, Figure 3).

**Table 5:** Relationship between male cognitive performance and female acceptance

Cognition measure	χ^*2*^	*df*	*p*	Adjusted *p*
**Inhibitory control**	**5.94**	**1**	**.015**	**.044**
PC1_learning_	1.82	1	.177	.531
Number of presentations to enter	4.25	1	.039	.118

We used generalized linear mixed models to examine the relationship between our three male cognition measures and female acceptance (entering a male's nest). Reported are the results of chi‐squared tests comparing a full model to a reduced model that did not contain the cognition measure as a fixed effect. Each model included seven covariates (male throat color score, eye intensity, nest area, courtship vigor, body mass, neophobia, and time to build a nest) and two random effects (male and female IDs). Significant effects after Bonferroni's correction are highlighted in bold.

## 4 DISCUSSION

This study was motivated by an interest in whether male sexual signals reflect their cognitive ability and whether cognitive ability is associated with measures related to male fitness. We first investigated whether male sexual signals (color, nest area, and courtship vigor) predict initial male performance on a detour‐reaching task, which is designed to assess inhibitory control (Hauser, 1999; Boogert, Anderson, et al., 2011; MacLean et al., 2014). As has often been found with problem‐solving performance (reviewed in Griffin & Guez, 2014), neophobia was a strong negative predictor of initial male detour‐reaching task performance. We included neophobia as a covariate in our statistical models such that we were asking to what extent sexual signals predict that aspect of performance on the detour‐reaching task that is independent of neophobia, which we interpret as inhibitory control. We found that no measured sexual signals (color, nest area, or courtship vigor) predicted initial detour‐reaching task performance after controlling for neophobia (i.e., inhibitory control, Tables 2, 3, 4). These sexual signals also did not predict measures of learning to solve the detour‐reaching task (Tables 2, 3, 4).

Females selected males who initially had better inhibitory control, after controlling for the effects of sexual signals (throat and eye color, nest area, and courtship vigor), body mass, neophobia, and time to build a nest (Table 5). The fact that residual variance in mating success is predicted by initial inhibitory control and that females did not observe males interacting with the detour‐reaching task, suggests females selected high performing males by assessing other traits not measured in this study (e.g., aspects of courtship behavior or other nest characteristics). This parallels findings in guppies; although female guppies preferred males who learned mazes quickly, learning speed was not associated with measured carotenoid‐based signals (Shohet & Watt, 2009).

Nest characteristics including the location (Candolin & Voigt, 1998), concealment (Sargent, 1982), and decoration (Östlund‐Nilsson & Holmlund, 2003) are examples of additional traits females may assess that might be related to male cognitive performance. Future studies could provide variation in nest site concealment opportunities and materials to determine whether these elements vary among males that differ in cognitive ability or whether males can learn aspects of nest construction (sensu Bailey, Morgan, Bertin, Meddle, & Healy, 2014). There are certainly other traits females may assess that were not tested in this study. For instance, plasticity in courtship behavior is important for mating success in other systems (Patricelli et al., 2002, 2006, 2011), and it likely requires cognitive skill to appropriately respond to diverse sets of females with different experiences and requirements (Keagy et al., 2009).

Finally, in this study, we were specifically interested in whether male cognitive ability at the time of mating (when females are choosing male mates who will father their offspring) was positively correlated with inhibitory control and/or learning a detour‐reaching task. For this reason, we measured both cognitive performance and sexual signals during the breeding season, such that we captured the sexual signals on which females base mating decisions. We speculate that inhibitory control is a critical trait for male sticklebacks who perform all the parental care but whose prey is similar in size and behavior to their own fry. Males with better inhibitory control may therefore be more likely to successfully raise their offspring to independence. It would be interesting to know whether cognitive performance varies between breeding and nonbreeding states throughout the year, particularly because major changes in reproductive state can affect both cognitive performance (Dunlap, Chen, Bednekoff, Greene, & Balda, 2006; Webster & Laland, 2011) and sexual signals (Kodric‐Brown, 1998). If inhibitory control is critical for male parental care, we would also predict that males have better inhibitory control as compared to females.

In summary, none of the male stickleback sexual signals we measured were predictive of initial inhibitory control or learning (as it relates to solving a detour‐reaching task). However, females preferred males with superior initial inhibitory control. Females in this experiment appeared to choose these males independently of sexual signals we measured, perhaps responding to male ability to tailor their courtship, although we did not test this hypothesis. Females could also get direct benefits from mating with males with better inhibitory control if these males are more likely to raise their offspring to independence.

## CONFLICT OF INTEREST

The authors declare no conflict of interest.
