# Supplementary material for: The relationship between male sexual signals, cognitive performance, and mating success in stickleback fish
Source: Ecol Evol. 2017 Jun 15;7(15):5621–31. doi: 10.1002/ece3.3091 (PMC5551085; doi:10.1002/ece3.3091)
Supplement: Supplementary file 1 [file ECE3-7-5621-s001.docx]

**Supplemental Table S1**

| N = 58 | Number of Trials to Solve | Change in Solves/ Attempts | Change in Time to Solve |
| --- | --- | --- | --- |
| Number of Trials to Solve | -- |  |  |
| Change in Solves/ Attempts | r = 0.23  p = 0.09 | -- |  |
| Change in Time to Solve | r = 0.14  p = 0.29 | r = -0.45  p = 0.0003 | -- |

**Correlation table for three measures of learning.** The two slope variables (change in solves/attempts and change in time to solve) were highly correlated with each other, but neither was correlated with number of trials to solve. Thus, we assessed number of trials to solve as a separate variable.
